# Exogenous Auxin and Gibberellin on Fluoride Phytoremediation by *Eichhornia crassipes*

**DOI:** 10.3390/plants12081624

**Published:** 2023-04-12

**Authors:** Lucas Rafael Lommez Vaz, Alisson Carraro Borges, Dimas Mendes Ribeiro

**Affiliations:** 1 Department of Agricultural Engineering, Federal University of Viçosa, Viçosa 36570-900, Brazil; 2 Department of Plant Biology, Federal University of Viçosa, Viçosa 36570-900, Brazil

**Keywords:** defluoridation, water hyacinth, accumulator plants, indole acetic acid, bioaccumulation, translocation

## Abstract

High rates of fluorosis were reported worldwide as a result of human consumption of water with fluoride contents. Adjusting fluoride concentration in water as recommended by the World Health Organization (<1.5 mg L^−1^) is a concern and it needs to be conducted through inexpensive, but efficient techniques, such as phytoremediation. The application of phytohormones was investigated as a strategy to improve this process. Thus, the main goal of this research was to evaluate the effect of exogenous auxin and gibberellin on the tropical duckweed *Eichhornia crassipes* performance for fluoride phytoremediation. Definitive screening and central composite rotatable designs were used for experiments where fluoride concentration (5~15 mg L^−1^), phosphorus concentration (1~10 mg L^−1^), and pH (5~9) were assessed as well throughout 10 days. Fluoride contents were determined in solution and plant tissues by potentiometry. Higher concentrations of fluoride reflected on greater absorptions by plants, though in relative terms removal efficiencies were quite similar for all treatments (~60%). Auxin and acidic conditions favored fluoride removals per mass of plant. Fluoride accumulated mostly in leaves and auxin probably alleviated toxic effects on *E. crassipes* while gibberellin showed no effect. Therefore, *E. crassipes* could be employed as a fluoride accumulator plant for water treatment and exogenous auxin may be used to improve the process.

## 1. Introduction

Fluoride (F^−^) presence in water is a modern challenge to overcome. Although low concentrations of fluoride in water were proven to benefit human health concerning dental well-being, elevated concentrations could lead to high rates of fluorosis and other diseases. This anion reaches fresh waters either through the weathering of geological material or via human activities, such as effluent disposal [1,2]. The World Health Organization (WHO) recommends a maximum value of 1.5 mg L^−1^ of fluoride in drinking water. However, many water sources present concentrations above this limit, especially groundwater, in which it is very common to observe concentrations on an average around 3 mg L^−1^ but not rarely above 20 mg L^−1^ [3,4], which is clearly a threat to public health [5,6].

Phytoremediation is a nature-based and cost-effective technique that combines plants and microorganisms, sometimes a filtering media, to treat water and wastewater [7,8,9]. Usually, this method employs macrophytes due to their great biomass production and robust metabolism, such as the giant duckweed *Eicchornia crassipes* [10,11,12]. As opposed to conventional technologies known to be expensive, highly mechanized, and complex (e.g., coagulation-precipitation, ion exchange, and membrane), phytoremediation gained attention for being environmentally friendly and for showing great efficiency towards a variety of contaminants, including those highly toxic and persistent, not to mention the potential for being applied in a decentralized manner [13,14]. Regarding fluoride, studies mostly look for accumulator species of plants that are tolerant to this contaminant [15,16]. Although fluoride is not essential to plants, they may uptake it from the soil, water, and air [17,18]. Plant fluoride absorption is related to the activity of this anion and to its speciation as well, which in turn is pH dependent [19]. *E. crassipes* was pointed to as a good defluoridation agent in recent reports [20].

Recent approaches to improve the performance of phytoremediation processes were reported, such as the use of growth promoting bacteria, transgenic plants, and phytohormones application. Plant hormones are low concentration molecules able to enhance plant metabolism and regulate growth, development, flowering, seed germination and dormancy, and many other processes [21,22,23,24]. Auxins and gibberellins are two very important groups of plant hormones. Auxins mainly influence cell elongation of stems and roots, apical dominance, and flowering, while gibberellins break seed dormancy, delay senescence, and induce various enzymes. Recent research involving the exogenous addition of phytohormones showed notable results to many contaminants [25,26,27,28,29,30]. It was suggested that these compounds alleviate phytotoxic effects caused by pollutants and may be able to enhance plant uptake of chemicals as well.

It is important to adjust fluoride water contents to recommended values using methods that are eco-friendly and cost-effective, such as phytoremediation. At the same time, there is plenty of room to search for improvements on this technique, such as the use of phytohormones. Thus, the main goal of this research was to evaluate the effect of exogenous auxin (IAA) and gibberellin (GA_3_) on *Eichhornia crassipes* performance for fluoride phytoremediation.

## 2. Materials and Methods

**Figure 1:**
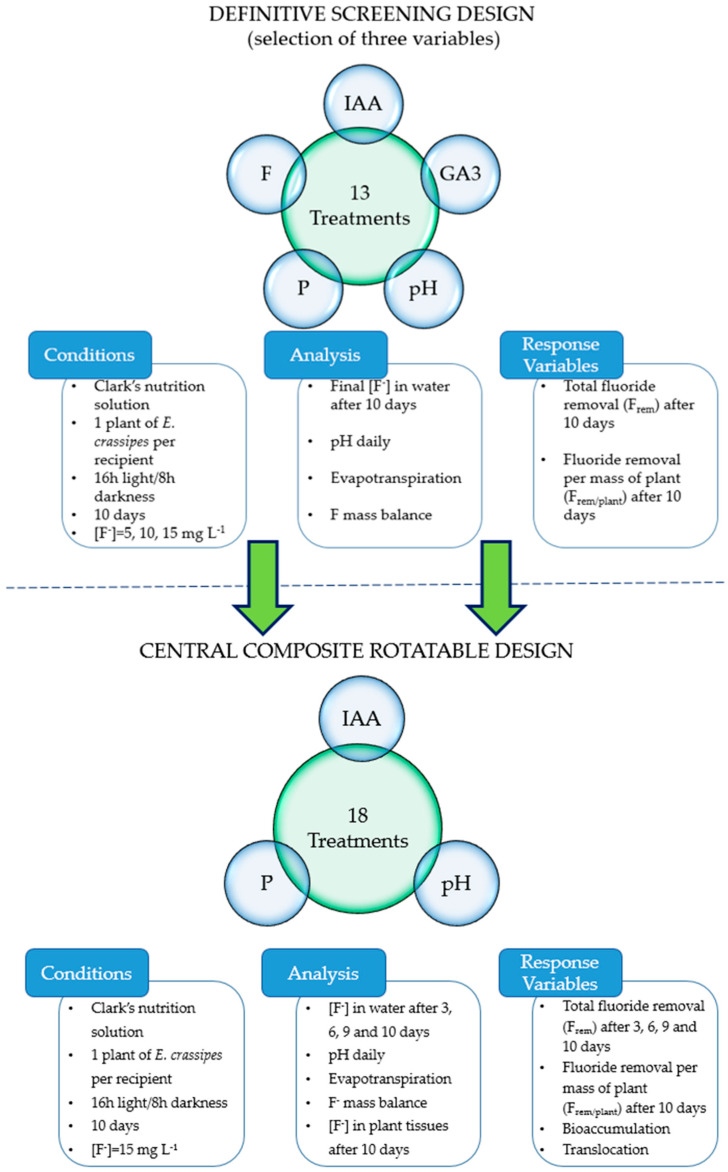
Flowchart of experimental set up presenting two subsequent experiments and their respective conditions, analyses, and response variables.

Two multivariate experiments were performed to assess the effects of five variables on the fluoride uptake by *E. crassipes*: auxin (IAA), gibberrelin (GA_3_), pH, phosphorus initial concentration, and fluoride initial concentration (Figure 1). A definitive screening design (DSD) (resolution IV) was used to select up to three variables that presented hierarchically greater influence on the phytoremediation process based on Pareto charts with standardized effects considering α at 10% and 5%. Similar to fractional factorial and Plackett–Burman designs, definitive screening designs are used to identify the most important factors that affect a certain process among a pack of factors considered too large, complex, or expensive to run every single combination between them. Screening experiments are usually followed by an optimization experiment that provides more detail on the relationships among the most important factors and the response variables. In our case, the three variables selected for further investigation were tested in a central composite rotatable design experiment (CCRD), a response surface design. Response surface designs are used to refine models after important factors using screening designs or factorial designs were identified; especially when curvature in the response surface is expected.

### 2.1. Definitive Screening Design Experiment

#### 2.1.1. Treatments and Design

For the DSD experiment, each of the five variables had three levels coded as −1, 0, and +1 as shown on Table 1. Acidic, neutral, and alkaline conditions were tested as pH values varied between 5 and 9. Initial fluoride concentrations simulated the most common concentrations reported for fresh water. Additionally, other reports showed that the activity of fluoride ions influenced the uptake for many plant species [19]. Phosphorus was selected, as this chemical is of high importance for plant health, and furthermore, it influences eutrophication processes of water bodies and fluoride precipitation. Phytohormones concentration values followed label recommendations and literature review. The absence of phytohormones was also tested as it was assigned a concentration of zero for the coded value of −1. The intention was to observe whether the simple presence of exogenous phytohormones (IAA, GA_3_) would actually make any difference to the remediation process.

Minitab software was used to create the treatments for the DSD experiment considering the adopted values for the variables (Table 2). One of these treatments represented the central point (T13) and each treatment had three replicates. Finally, two extra recipients were used as blank treatments for control means, totaling 41 recipients.

#### 2.1.2. Collection and Acclimation of *Eichhornia crassipes*

The *E. crassipes* plants were collected in early stages of development at one of the botanic gardens of the Federal University of Viçosa, Brazil. They were washed with sodium hypochlorite solution and thoroughly rinsed with distilled water. Then, they went through acclimation for 30 days in Clark’s nutrient solution [31] at pH 6.5. Clark’s solution consisted of 6.9 mM N-NO_3_, 0.9 mM N-NH_4_, 0.0069 mM P, 1.8 mM K, 2.6 mM Ca, 0.6 mM Mg, 0.5 mM S, 0.5 mM Cl; 0.007 mM Mn, 0.019 mM B, 0.002 mM Zn, 0.0006 mM Mo, 0.0005 mM Cu, and 0.038 mM Fe-EDTA. Plants were kept at room temperature and artificial light with a photoperiod of 16 h of light and 8 h of darkness controlled with a timer. Lighting structure was built with white fluorescent tube lamps (32 mm × 1212 mm, 40 W, 2650 lm, and 41.6 µmol m^−2^ s^−1^) at 60 cm above plants. These conditions were maintained during the experiments as well.

#### 2.1.3. Experimental Conditions

After acclimation, plants were transferred to individual polyethylene recipients (25 cm of diameter) containing 2 L of Clark’s solution. Then, the combination of factors to each recipient was developed according to the treatment they represented. Considering that *E. crassipes* has a high relative growth rate, as well as a very robust metabolism, a period of 10 days was enough to assess its fluoride uptake from water. Recipients were randomly distributed under the lighting structure and they were shuffled once again at day 5. For control reference, two other recipients were also placed under the light; one of them with 15 mg L^−1^ of fluoride but with no plant, and the other with a plant but no fluoride (0 mg L^−1^).

Fluoride was added to each treatment as sodium fluoride (NaF), while phosphorus was added as calcium phosphate monobasic (Ca(H_2_PO_4_)_2_.H_2_O); pH was adjusted with hydrochloric acid (HCl) and sodium hydroxide (NaOH). Auxin was added directly to the solutions of each recipient, while gibberellin was added by spraying. These chemicals were all Sigma Aldrich products.

#### 2.1.4. Analyses

The pH values were monitored daily with a multi parameter water quality meter (HQ30d Hach IntelliCAL^TM^ PHC 101) and gathered in graph to present central tendencies for each treatment.

Final fluoride concentration was determined with an ion selective electrode (ISE), method 4500-F^−^ C of *Standard Methods* [32] after 10 days of treatment. The ISE is solid-state and based on a lanthanum fluoride monocrystal (LaF_3_). The reference electrode consists of Ag/AgCl with a double junction, with an external solution of 10% *w/v* NaNO_3_ and an internal solution of 3 mol L^−1^ KCl saturated with AgCl. Evapotranspiration was determined using the difference between initial and final volume after 10 days for each recipient. The average evapotranspiration was considered for mass balance calculations and fluoride removal ($F_{rem}$, mg) (Equation (1)), where ${\left[F^{-}\right]}_{i}$ and ${\left[F^{-}\right]}_{f}$ are initial and final concentrations of fluoride (mg L^−1^), respectively, and $V_{i}$ and $V_{f}$ are initial and final volume (L), respectively. These results were also expressed in percentage (Equation (2)). Additionally, even though plants in apparent same conditions were selected (e.g., weight, size, and number of leaves); the fluoride removal per dry mass of plant ($F_{rem/plant}$, mg g^−1^) was also taken into consideration (Equation (3)) at the end of the experiment to equalize the results even more, where M represents the dry mass of plant (g). It is important to state that the results achieved with Equation (3) do not represent bioaccumulation factors since the analyses did not occur using plant tissues.

(1) $$F_{rem}={\left[F^{-}\right]}_{i}\cdot V_{i}-{\left[F^{-}\right]}_{f}\cdot V_{f}$$

(2) $$F_{rem}(\%)=\frac{{\left[F^{-}\right]}_{i}\cdot V_{i}-{\left[F^{-}\right]}_{f}\cdot V_{f}}{{\left[F^{-}\right]}_{i}\cdot V_{i}}\times 100$$

(3) $$F_{rem/plant}=\frac{{\left[F^{-}\right]}_{i}\cdot V_{i}-{\left[F^{-}\right]}_{f}\cdot V_{f}}{M}$$

Results were evaluated using Pareto charts developed by Minitab18 in order to identify the factors that had a significant effect on the response variables ($F_{rem}$ and $F_{rem/plant}$) and to what extent these effects were considering α at 5% and 10%. A model was obtained for the observed data for the response variable $F_{rem/plant}$. The model was analyzed in regards to the coefficient of determination (R^2^) and residuals. Contour plots were also generated for factors in pairs to assess the behavior of response variables and identify tendencies provided by the variation in a pair of factors, while others were constant, which allowed for establishment of the range of variables for the following experiment. Three variables remained for the next assay in a central composite design (CCRD).

### 2.2. Central Composite Rotatable Design Experiment

#### 2.2.1. Treatments and Design

A central composite design is a response surface methodology used to estimate curvature without needing complete factorial experiments. With the results obtained after the definitive screening design experiment, three variables were selected for further evaluation in a CCRD experiment: auxin, phosphorus, and pH. Each variable had five levels coded as −α, −1, 0, 1, and α, as shown on Table 3, where α = 1.682. Levels were determined after establishing the range of the variables based on results observed on a previous DSD experiment.

Minitab software for statistical analyses was used to create the 18 treatments for the CCRD experiment considering the adopted values for the variables (Table 4). Four of these treatments represented the central point (T15, T16, T17, and T18) and each of the other 14 treatments had two replicates. Finally, two extra recipients were used as blank treatments for control reference, totaling 34 recipients.

#### 2.2.2. Experimental Conditions

The same procedure described for the DSD experiment was used; so one plant per recipient containing 2 L of Clark’s nutrition solution and the combination of factors for each treatment. Treatments were randomly distributed under the artificial light and monitored for 10 days. They were shuffled one more time at day 5.

This time, based on results observed in the DSD experiment, all plants were exposed to a fluoride concentration of 15 mg L^−1^ in order to assess their performance in the most severe scenario among the ones tested herein. For control reference, two other recipients were also placed under the light, one of them with 15 mg L^−1^ of fluoride but with no plant and the other with a plant but no fluoride (0 mg L^−1^). Fluoride and phosphorus were added as sodium fluoride (NaF) and calcium phosphate monobasic (Ca(H_2_PO_4_)_2_.H_2_O), respectively. The pH was adjusted with hydrochloric acid (HCl) and sodium hydroxide (NaOH). Lastly, auxin was added directly to the solutions.

#### 2.2.3. Analyses

The pH was monitored daily. Samples of 15 mL were taken to determine fluoride concentration at day 3, 6, 9, and 10 (final) using an ISE [32]. Instruments and chemicals were the same as previously mentioned for the DSD experiment. $F_{rem}$ (Equation (1)), $F_{rem}(\%)$ (Equation (2)), and $F_{rem/plant}$ (Equation (3)) were employed to register results, considering the first two for days 3, 6, 9, and 10, and the latter only at the end of experiment (10 days).

Fluoride contents were also measured in plant tissues at the end of the experiment (10 days) using extraction procedures in order to determine bioaccumulation and translocation factors. Roots and aerial parts were analyzed separately. They were oven dried at 65 °C for 72 h and then weighted, grinded in ball mill, and sieved (20 mesh). Samples of 0.25 g were added to 50 mL falcon tubes containing 5 mL of sulfuric acid (H_2_SO_4_) 1 N. Tubes were placed in ultrasonic bath (LGI-LUC-240) at 50 °C for 20 min. Later, 5 mL of sodium hydroxide (NaOH) 1 N were added to each falcon tube followed by a 10 mL addition of total ionic strength adjusting buffer (TISAB) solution [32], totaling a volume of 20 mL per sample. Finally, samples were analyzed with the ISE. Readings in millivolt were converted to fluoride concentrations that subsequently were converted to fluoride mass contained in the extraction volume of 20 mL obtained from 0.25 g of dried plant material. Results were extrapolated to the total dried mass of each plant.

Bioaccumulation (BF) and translocation (TF) factors were calculated as shown in Equations (4) and (5) [16], respectively. BF was determined considering the total dry mass of each plant. Fluoride contents in whole plants ($M_{F-plant}$) were divided by the remaining fluoride mass in respective solution ($M_{F-sol}$), both in mg. As for the TF, the fluoride concentration registered for the aerial parts (${\left[F^{-}\right]}_{aerial}$) was divided by the concentration found in roots (${\left[F^{-}\right]}_{root}$), both in mg g^−1^ (dry weight).

(4) $$BF=\frac{M_{F-plant}}{M_{F-sol}}$$

(5) $$TF=\frac{{\left[F^{-}\right]}_{aerial}}{{\left[F^{-}\right]}_{root}}$$

A stacked column chart was generated to present the accumulated fluoride removals throughout the experiment on days 3, 6, 9, and 10. Results were evaluated using Pareto charts developed by Minitab18, as well as analyses of variance (ANOVA) for fluoride removal ($F_{rem}$) and fluoride removal per dry mass of plant ($F_{rem/plant}$) for α at 5% and 10%. A regression analysis considering a full quadratic model was performed for the response variable $F_{rem/plant}$_,_ and an equation to describe results was obtained. Non-significant terms were removed when necessary to reduce and refit the obtained model. The model was analyzed in regards of coefficient of determination (R^2^) and residuals. Contour plots and a surface plot were also generated for factors in pairs to assess the behavior of the response variable $F_{rem/plant}$ and identify tendencies provided by the variation in a pair of factors, while the other remained constant at the optimum value.

## 3. Results

### 3.1. Definitive Screening Design

Table 5 shows the experimental results of the DSD. In a preliminary view, treatments that contained more fluoride were the ones where greater masses of this anion were removed. However, in relative terms (%), all treatments performed quite similar to one another, as values remained between 54.9 and 63.0%.

**Figure 2:**
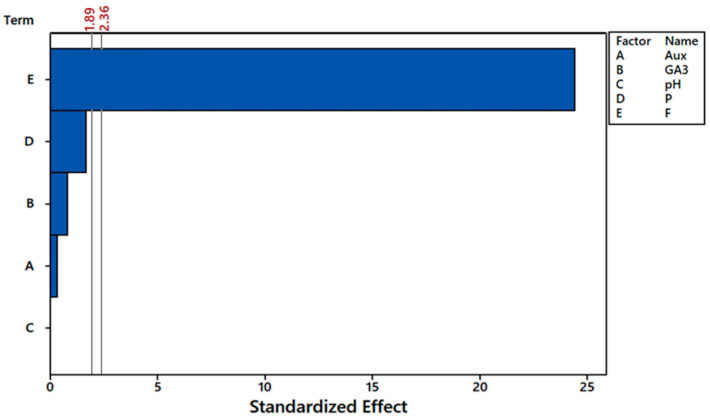
Pareto chart of first order effects for auxin (A), gibberellin (B), pH (C), phosphorus initial concentration (D), and fluoride initial concentration (E) on removed mass of fluoride, considering α = 10% (1.89) and α = 5% (2.36). The bars behind the thresholds of α values represent factors that were not significant to the response variable.

The fluoride initial concentration had the greatest effect on fluoride mass removal results, remarkably higher than the other variables (Figure 2). In fact, it was the only factor statistically significant for α = 5%. The other variables stayed behind the threshold for statistical difference (2.36) even for α = 10% (1.89) at the Pareto chart.

**Figure 3:**
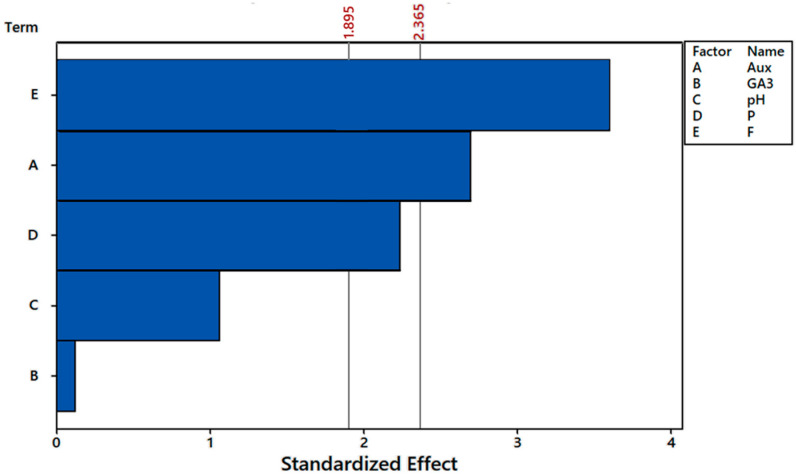
Pareto chart of first order effects for auxin (A), gibberellin (B), pH (C), phosphorus initial concentration (D), and fluoride initial concentration (E) on fluoride removal per dry mass of plant, considering α = 10% (1.895) and α = 5% (2.365). The bars behind the thresholds of α values represent factors that were not significant to the response variable.

As for the fluoride removal per dry mass of plant, the fluoride initial concentration and auxin showed an effect for α = 5% and phosphorus initial concentration for α = 10%, whereas pH and gibberellin were not significant (Figure 3). The obtained model fit quite reasonably to the observed data as R^2^ = 79%. Additionally, the quality of the model was certified, considering that the residuals are randomly distributed and have constant variance. However, models at screening experiments should be used with caution, and the aforementioned parameters (R^2^ and residuals) were assessed to assure the results’ reliability.

For gibberelin, fluoride removals were increased by an application of 25 µM GA_3_ as compared with the control (0 µM GA_3_), even though such an occurrence was not statistically significant (10%). Interestingly, treatment of *E. crassipes* with 50 µM GA_3_ promoted a similar increase in fluoride removals in relation to the 25 µM GA_3_, indicating that fluoride removal response to 50 µM GA_3_ is saturated. Thus, there should not be increments in fluoride removals by *E. crassipes* plants when supplemented with GA_3_ at doses above 25 µM. Additionally, there was an increase in fluoride removal in plants treated with IAA, indicating that this treatment had an effect on fluoride uptake.

**Figure 4:**
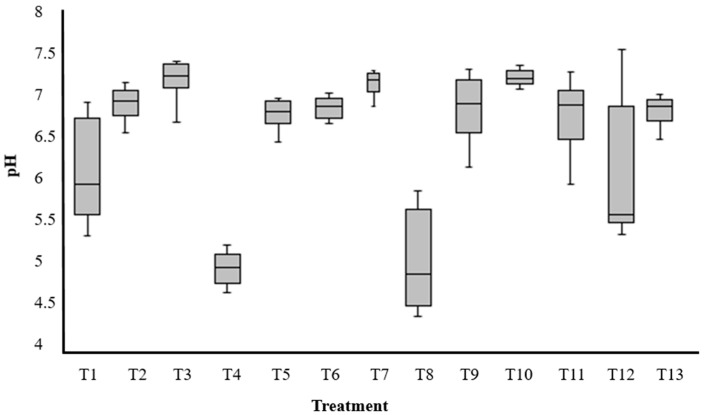
Boxplot graph of pH values daily collected from treatments 1 to 13 throughout 10 days on the definitive screening design experiment.

During the 10 days of experiment, all pH values ranged between 4.4 and 7.5 (disregarded initial values) and 70% of them remained between 6.5 and 7.5 (Figure 4). Therefore, plants helped to bring pH close to neutral values to the majority of treatments for a considerable part of this 10-day period.

Conclusively, it was very clear that fluoride initial concentration was deeply involved in the amounts of fluoride accumulated by *E. crassipes*, considering both response variables ($F_{rem}$ and $F_{rem/plant}$), which corroborate the observation of other reports testing different plants exposed to fluoride. Due to this query settlement, it was decided not to follow through with this variable to the next CCRD experiment, even though it was the one with the greatest effect. It is suggested that future evaluations work this variable individually, i.e., in a univariate manner, expanding the interval of concentrations to find a point at which increments on fluoride absorption by *E. crassipes* can no longer be seen due to the unbearable phytotoxic effects of the excess of this contaminant. As a reminder, water sources presenting fluoride concentrations above 15 mg L^−1^ exist but do not represent the majority, except when such sources are used for industrial wastewater disposals. In this way, *E. crassipes* was clearly shown to be tolerant to the concentrations tested that represent the majority of water sources. Because of that, it was decided to further test the plant’s performance at the most severe scenario among the concentrations tested, i.e., the CCRD experiment occurred considering a fluoride concentration of 15 mg L^−1^ for all treatments.

Proceeding with the sequence of variables that affected fluoride removal per dry mass of plant (Figure 3), it remains the phytohormone auxin, phosphorus initial concentration, and pH, though the latter was seen not to have a significant effect (α = 10%) within the tested range (5~9). These variables were taken to experiment in a central composite rotatable design. GA_3_ presented the least effect on $F_{rem/plant}$, and therefore was not taken to the next assay.

**Figure 5:**
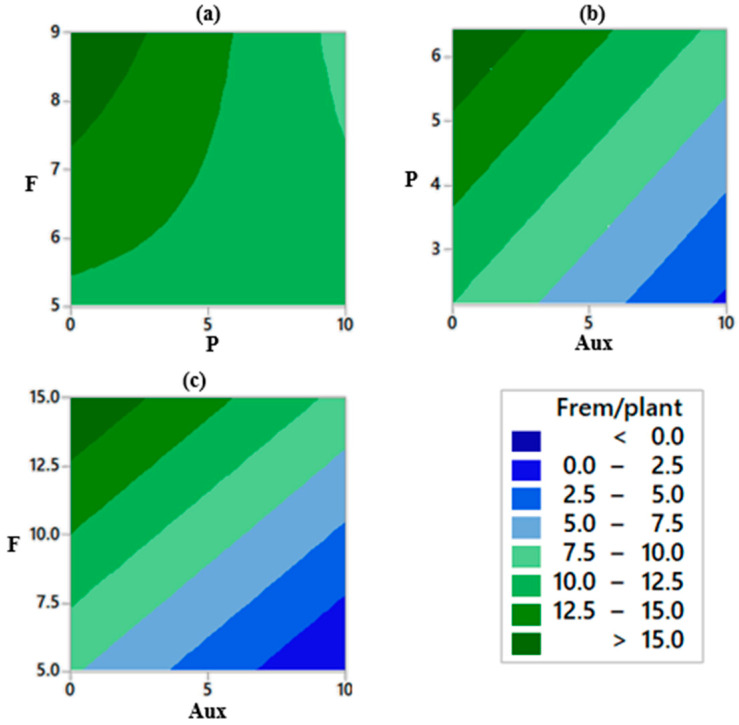
Contour plots for fluoride removal per dry mass of plant. Each plot represent a pair of variables: (**a**) P x F; (**b**) Aux x P; and (**c**) Aux x F. Note: pH and GA_3_ were not represented once no significant effect was detected (α = 10%). For each pair of variables represented in one graph, the other three were held on the values coded as zero (medium), hence Aux = 5 µM, GA_3_ = 25 µM, pH = 7, P = 4.28 mg L^−1^, and F = 10 mg L^−1^.

Concerning auxin doses, the greatest fluoride removals per dry mass of plant occurred below 5 µM, more specifically below 3 µM, represented by darker shades of green on Figure 5. High concentrations of sinthetic auxins may provide toxic effects on plants, especially dicotyledonous plants. Fluoride and phosphorus initial concentrations, F and P on Figure 5, respectively, did result in greater fluoride removals as these variables increased. Contour plots really show the potential of even greater fluoride removals in case the test region is extended to higher values for both variables, i.e., above 15 mg L^−1^ for F and 6.42 mg L^−1^ for P.

Therefore, based on the DSD results, the CCRD experiment was planned to further evaluate the effect of auxin, phosphorus initial concentration, and pH, where the interval of these variables was restricted, expanded, and maintained, respectively.

### 3.2. Central Composite Rotatable Design

Trends detected on the DSD experiment were considered to establish the range of the variables for the CCRD experiment. Auxin, which previously varied between 0 and 10 µM, was narrowed to the interval of 0 to 2.5 µM, since higher fluoride removals were seen below 3 µM (Figure 5). As for phosphorus initial concentration, the experimental region was expanded at both ends and varied between 1 and 10 mg L^−1^. Finally, pH remained between 5 and 9. All plants were exposed to a fluoride concentration of 15 mg L^−1^ as it was noted that *E. crassipes* fairly tolerated such contamination levels.

**Figure 6:**
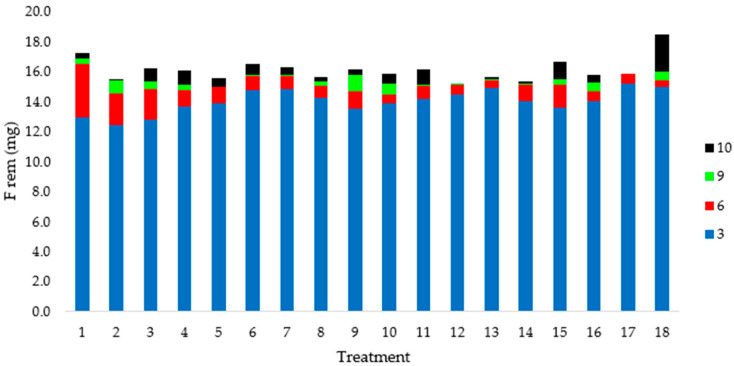
Increments of fluoride removals (mg) until day 3 (blue), 6 (red), 9 (green), and 10 (black) for treatments 1–18 on the CCRD experiment.

Fluoride removals were assessed throughout 10 days and samples of days 3, 6, 9, and 10 were taken for analyses. Removals majorly occurred on the first 6 days after exposure (84 to 99%) and were particularly intense up until day 3 for all treatments (Figure 6). From day 6 on, increments on fluoride removals were quite discreet. Other reports reached similar results.

**Figure 7:**
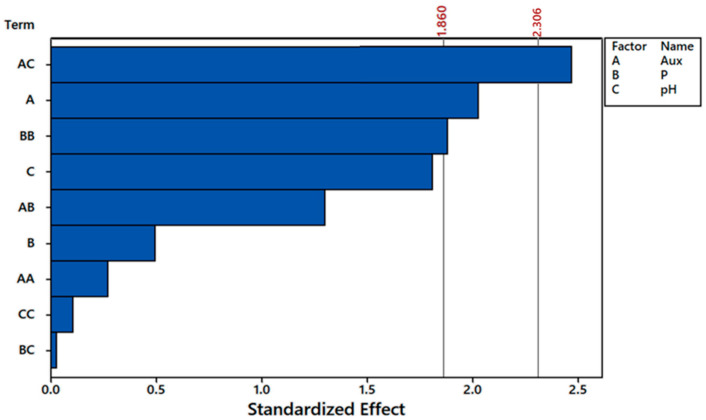
Pareto chart of full quadratic model at α = 10% (1.860) and α = 5% (2.306) for fluoride removal per dry mass of plant in the CCRD experiment. Auxin (A), phosphorus initial concentration (B), and pH (C). The bars behind the thresholds of α values represent factors that were not significant to the response variable.

Total fluoride removals varied from 49.4 to 61.8% after 10 days (Table 6), indicating that *E. crassipes* presents a great capacity for fluoride remediation. No trends were detected regarding the effects of auxin, phosphorus, or pH over fluoride removal (F_rem_) (Table 6). The analyses of variance did not indicate significant effects for any factor (α = 10%) on this response variable. On the other hand, the best results for fluoride removal per dry mass of plant (F_rem/plant_) occurred on treatments T13, T2, and T10, where values surpassed 6 mg g^−1^. Such removals were reached in acid to neutral pH conditions and higher doses of auxin. In fact, the interaction between these two variables was significant to α = 10% and α = 5% (Figure 7).

**Figure 8:**
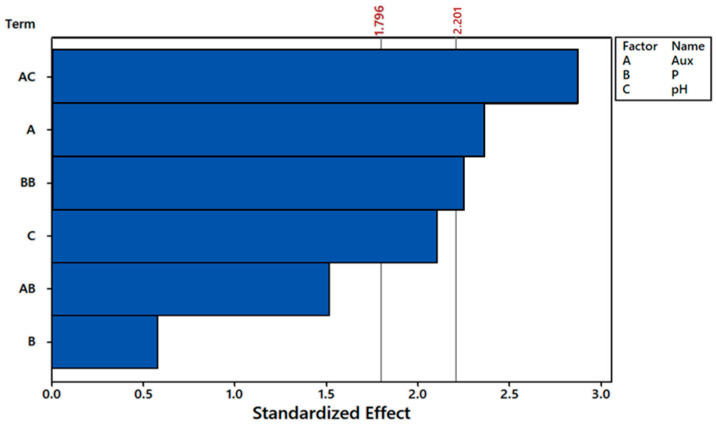
Pareto chart for fluoride removal per dry mass of plant in CCRD experiment at α = 10% (1.796) and α = 5% (2.201), disregarding BC, CC, and AA terms from full quadratic model. Auxin (A), phosphorus initial concentration (B), and pH (C). The bars behind the thresholds of α values represent factors that were not significant to the response variable.

Full quadratic model analyses indicated that not only the interaction between auxin and pH was significant for α = 10%, but also the first-order effect of auxin (A) and second-order effect of phosphorus (BB) (Figure 7). All the other terms did not influence fluoride removal per dry mass of plant ($F_{rem/plant}$), so in order to simplify the model, three of the least significant terms were dismissed, i.e., AA, CC, and BC (Figure 8). By doing so, terms that were once significant at α = 10% (AC, A e BB) became significant at α = 5%. Moreover, the first-order effect of pH (C) became significant at α = 10%.

**Figure 9:**
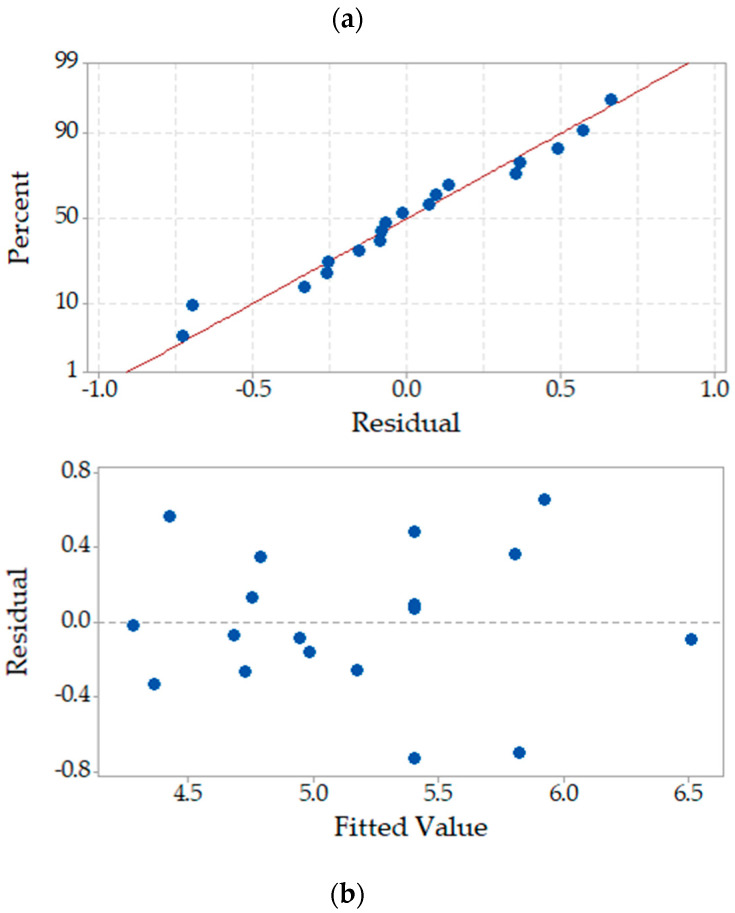
Residual plots for fluoride removal per dry mass of plant. (**a**) Normal probability plot of residuals and (**b**) residuals versus fits. Residuals are randomly distributed and have constant variance.

Model (Equation (6)) was significant at α = 5% (*p*-value = 0.018) and presented R^2^ = 70.2%, which is quite reasonable. The lack of fitting was not significant (*p*-value = 0.605), meaning that the generated model did fit the observed data. Homoscedasticity analyses indicated that residuals are randomly distributed and have constant variance (Figure 9).

(6) $$F_{rem/plant}=-0.43+5.08Aux+0.592P+0.468pH-0.0415P\cdot P-0.1319Aux\cdot P-0.562Aux\cdot pH$$

**Figure 10:**
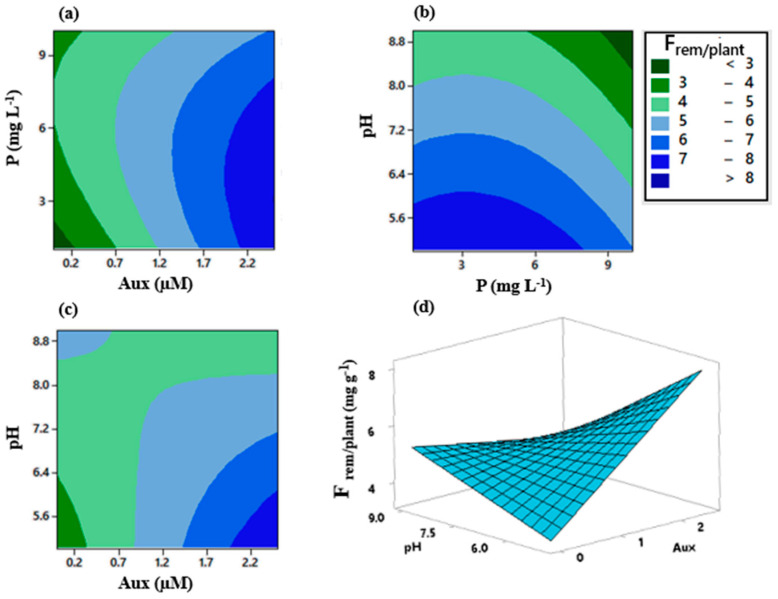
Contour and surface plots for fluoride removal per dry mass of plant. Each plot represents a pair of variables: (**a**) Aux x P; (**b**) P x pH; (**c**) Aux x pH; and (**d**) Aux x pH. Note: For each pair of variables represented in one graph, the other variable was held on optimum value, hence Aux = 2.5 µM, pH = 5, and P = 3.2 mg L^−1^.

As it was previously mentioned, greater fluoride removals per dry mass of plant ($F_{rem/plant}$) were observed in acid to neutral pH conditions and higher doses of auxin that could also be seen by contour plots (Figure 10). Furthermore, the interaction between these two factors was significant along with the first-order effect of auxin (α = 5%). Exogenous adding of this phytohormone potentially improved *E. crassipes* tolerance to phytotoxic effects of fluoride as well as enhanced fluoride uptake, as was also reported in other studies. As for the other terms, although it is possible to notice a gradient for greater fluoride removals per dry mass of plant on the phosphorus-auxin and pH-phosphorus plots, the interactions between these variables were not significant considering ANOVA results. However, phosphorus did present a significant second-order effect (α = 5%), demonstrating that this variable induced curvature to the response variable dataset.

Optimum values for auxin, phosphorus, and pH from the model obtained for fluoride removal per dry mass of the plant (Equation (6)) were 2.5 µM, 3.2 mg L^−1^, and 5, respectively. For these values, $F_{rem/plant}$ reached 8 mg g^−1^. When pinning optimum values for two of the three variables, $F_{rem/plant}$ remains above 6 mg g^−1^ for auxin between 1.4 and 2.5 µM, for pH between 5 and 7.1, and for the entire interval of phosphorus, i.e., 1 to 10 mg L^−1^.

After 10 days of fluoride exposure, bioaccumulation factors were, on average, 0.114 ± 0.038. Minimum and maximum occurred for treatments T2 and T1, respectively (Table 7), where for each milligram of fluoride remaining in the solution, there was 0.042 and 0.201 milligrams of fluoride in the plants of these treatments, respectively, as well. Translocation factor varied from 0.655 to 2.374, in general more than 1 (Table 7). Hence, there was a greater accumulation of fluoride in aerial parts of the plants than in roots.

**Figure 11:**
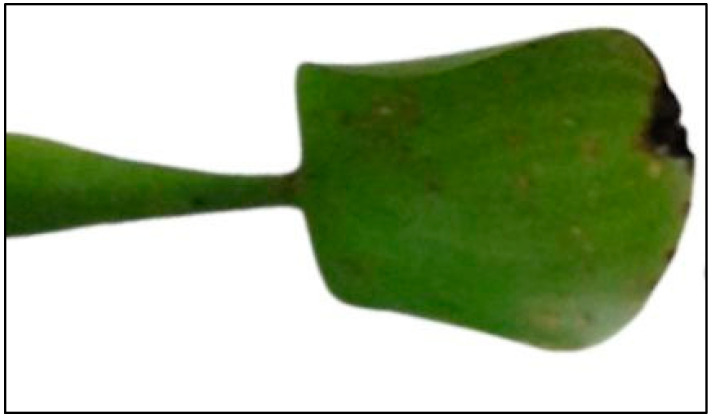
*Eichhornia crassipes* leaf presenting necrosis on margin and apex due to the exposure to 15 mg L^−1^ of fluoride for 10 days.

The effects of the accumulation and translocation of fluoride were noticed through damages inflicted on leaves. Visual symptoms of 15 mg L^−1^ of fluoride exposure included necrosis on leaf margin and apex (Figure 11).

## 4. Discussion

Fluoride uptake varies a lot depending on plant species, considering this ion is not essential to plants. The concentrations to which they are exposed play an important role as well [19,33]. A positive correlation between fluoride accumulation and fluoride concentration was also observed for *Avena sativa* and *Lycopersicon esculentum* [19]; *Camellia sinensis* [34]; *Linaria vulgaris* and *Mentha arvensis* [33]; *Olea europaea* [35]; *Camellia japonica* and *Saccharum officinarum* [36]; *Pistia stratiotes* and *Eicchornia crassipes* [15]; and *Pistia stratiotes* [37]. For Khandare et al. [16], although downward percentages of fluoride accumulation were presented as concentrations went up, the removal of fluoride in absolute terms by *Nerium oleander* actually increased as well. Karmakar et al. [37] registered higher absorptions of fluoride by *P. stratiotes* and *E. crassipes* as concentration increased, but the same could not be seen for *Spirodela polyrhiza*. Kostyshin et al. [33] also observed the same occurrence for many species on their evaluation.

The fluoride concentrations that plants can tolerate are highly dependent on plant species. Some species are more sensitive to phytotoxic effects of this contaminant and cannot survive or remain healthy even when exposed to small concentrations. On the other hand, tolerant species can thrive in highly contaminated environments. The results found herein for *E. crassipes* indicate that fluoride uptake was higher when concentration increased, quite similar to many other reports testing different species. However, there should be a threshold to this phenomenon where any plant would not increment fluoride absorption due to the severe phytotoxic effects of the environment. Nevertheless, the most common concentrations of fluoride in water sources could not be considered inhospitable for *E. crassipes* based on our results.

Exogenous application of plant hormones was investigated as a possibility to enhance the phytoextraction of contaminants, mostly metals, and improve tolerance to their toxic effects on plants. Reports regarding hormones providing these effects can be seen not only for auxin [25,26,27,28,38,39] and gibberellin [29,30,40], but also for other hormones and compounds, such as cytokin, salicilic acid, jasmonic acids, ethilene, abscisic acid, and so on [21,38,41,42,43]. In our case, auxin provided greater fluoride removals, while gibberellin had no effect, which could be related to the intervals adopted or the type of application (spraying). Auxin also helped *E. crassipes* in biomass gains, as it was the only variable to present significant effect (α = 5%) to this response variable, and provided a higher bioaccumulation factor in comparison to other findings [15], which could be a sign of toxicity alleviation for the plants. Thus, the auxin supplementation potentially improved *E. crassipes* tolerance to fluoride phytotoxic effects and facilitated the uptake of this contaminant, as also seen in other reports assessing lead [25,26,27,38], cadmium [28,39], and zinc [26]. It is important to mention, though, that best results were found at doses below 3 µM (Figure 5, DSD experiment). High concentrations of sinthetic auxins may provide toxic effects on plants, especially dicotyledonous plants. In fact, these hormones are even used as herbicides [44].

Auxins have an important role in plant growing and stress defense, and they were shown as promising on metal phytoextraction, though the mechanisms through which these processes happen are yet to be fully cleared [28,45]. Considering the results herein observed, it is suggested that the effect of such hormones may also apply to situations where stress is caused by a non-metal contaminant; in this case, fluoride. Under abiotic stress, auxin inhibition is likely to occur, but such a hormone is exactly one of the tools plants have to deal with stresses. Hence, exogenous addition of auxin may prevent potential damages that an adverse media would inflict, helping plants to thrive beyond such circumstances [45]. For example, exogenous auxin (IAA) reverted inhibitory effects of NaF on *Avena sativa* [46].

In general, pH remained between 6.5 and 7.5 during the DSD experiment. Plants helped to bring pH close to neutral values to the majority of treatments for a considerable part of this 10-day period. When potentially toxic elements are present in the media, plant roots may act to diminish toxicity through the release of exudates capable of operating pH changes [47]. The pH of the solution has a direct influence on the amount of fluoride ions that are bioavailable to adsorption and absorption processes around the root zone [19,37]. It was pointed out that pH is very peculiar on the sites where plants normally accumulate fluoride ions, as this storage tends to occur in organelles with an alkaline environment, such as mitochondria [30]. The acid to neutral pH conditions favoring fluoride removals in CCRD experiment was also observed by other reports with *Pistia stratiotes* [37] and *Camellia sinensis* [34]. In slightly acid conditions, fluoride anions tend to pair with free H^+^ cations, forming HF, which is easily absorbed by plants [48]. A model describing the phytoremediation of fluoride by *Landoltia punctata* generated optimum removals at both acid and alkaline pH conditions [49]. In alkaline solutions, the anion F^−^ is predominant and more bioavailable for root absorption. As fluoride absorption is positively correlated with this anion activity, at least up until non phytotoxic amounts, the higher the bioavailability in alkaline solutions, the greater the removals [19,33,50].

In regards to phosphorus, *Landoltia punctata* was influenced by this element during fluoride phytoremediation, in which greater amendments of phosphorus provided higher fluoride removals [49]. Phosphorus is a macronutrient deeply involved in plant development and metabolism [51,52,53]. As seen on the screening assay, phosphorus influenced fluoride removals providing greater efficiencies. Fluoride and phosphorus, the latter in phosphate form, do not compete for the same absorption sites on plant roots [19]. The deprivation of phosphorus, however, reduces plant growth limiting transpiration rates and water uptake that in turn would decrease fluoride absorption as such ion enters roots through passive processes [51,52,53]. The second-order effect observed on the CCRD assay potentially occurred due to the expansion of the interval tested, in which this time, higher concentrations of phosphorus reduced defluoridation. Phosphorus presence potentially contributed to some fluoride precipitation in the form of fluorapatite (Ca_5_(PO_4_)_3_F), a process that in fact was used and researched as another defluoridation technique [54,55].

Considering fluoride contents in plant tissues (mg g^−1^), the results herein observed correspond to those reported for *Olea europaea* in pot experiments with soil spiked with fluoride [35] and are rather superior to those found for *Pistia stratiotes*, *Spirodela polyrhiza,* and *Eichhornia crassipes* in hydroponic studies [15]. Specifically for *E. crassipes*, the authors observed fluoride contents of 0.038; 0.092; 0.172; and 0.214 mg g^−1^ (dry weight) for an initial concentration of 3, 5, 10, and 20 mg L^−1^, respectively, considering the whole plant. In our case, for an initial concentration of 15 mg L^−1^ of fluoride, the contents found in whole plants varied from 0.59 to 1.22 mg g^−1^ (dry weight). This is a sign that the addition of exogenous auxin contributed to greater removals of fluoride from solution. On the other hand, Khandare et al. [16] achieved higher bioaccumulation values for *N. oleander*, *P. crinitum*, and *P. olearacea*, after exposure to 10 mg L^−1^ of fluoride.

Fluoride accumulation occurred mostly on aerial parts of the giant duckweed making translocation factors be greater than 1. This was also observed for *Camellia japonica* (1.3 to 2.7) [36]; for *N. oleander* (1.85), *P. crinitum* (1.19) e *P. olearacea* (1.43) [16]; and for *Camellia sinensis* [34], but other plants were reported to accumulate greater amounts of fluoride in the roots [35,36,56,57]. Such accumulations occurred mostly in the first 6 days of exposure, as also reported in other investigations. *Hydrilla verticillata* exposed to 3–20 mg L^−1^ [58] and *Pistia stratiotes* exposed to 1–25 mg L^−1^ of fluoride [37] did not accumulate relevant amounts of the anion after 7 and 8 days of exposure, respectively. Absorption rates for the latter were especially intense until day 4. For *Camellia japonica* it was observed decreasing fluoride concentrations in solution for 18 consecutive days when concentration started out as 2.5 mg L^−1^, while for concentrations of 5 and 10 mg L^−1^, reductions were drastic on the first 3 days and further on stayed minimal [36]. Plants can accumulate contaminants in a relatively intense fashion on the first days of exposure and then reduce and stabilize absorption rates due to the saturation of accumulation sites and due to the effects caused by the amounts already stored in their tissues [30]. In addition, since concentration and ion activity have an influence on uptake rates, the gradual fluoride absorptions lead to less contaminant available in solution, even though some water was in fact lost in the process [19,33]. This is a limitation of hydroponic studies where solution is not renovated throughout the tests. Furthermore, this indicates a concern for phytoremediation treatment systems (e.g., constructed wetlands) where plants need to be constantly replaced or have their propagation rates monitored, otherwise efficiencies tend to decrease with time.

The effects of fluoride accumulation on *E. crassipes* plants did not go unnoticed as visual symptoms could be seen on leaf margins and apex as a result of greater accumulation on aerial parts (i.e., TF > 1) in general. Necrosis and chlorosis on leaves are usually the first and more visible symptoms of fluoride exposure. Another morphological disturbance often detected is the reduction in leaf area, which in turn affects biomass production [17,59,60]. However, the translocation factor, as well as bioaccummulation and fluoride distribution in plant tissues and organs, varies a lot depending on the species and age of plants [34,35,61,62]. These processes are also influenced by the phase of the plant cycle, season, and ultimately even between varieties of the same species [59]. Furthermore, they are dynamic processes considering contaminants may enter (influx) and exit (efflux) a certain living organism through various pathways instead of being indefinitely stored in tissues [63]. Therefore, other species of plants were reported to accumulate more fluoride in the roots rather than aerial parts, i.e., with translocation factors less than 1, which was claimed to be the greater tendency [64]. *Saccharum officinarum* [36], *Olea europeae* [35], *Brassica juncea* [56], and *Prosopis juliflora* [57] followed this trend. Nevertheless, it is safe to say that *E. crassipes* was shown to be tolerant to the fluoride concentrations that they were exposed to in this study and performed quite well on fluoride phytoremediation considering the circumstances tested.

In comparison to other hydroponic studies, *Saccharum officinarum* was considered good for remediation after reaching up to 40% of fluoride removals for the initial concentration of 4 mg L^−1^ [65]. For fluoride concentrations ranging from 3 to 20 mg L^−1^, *P. stratiotes* was able to remove between 15 and 24% from water, while *S. polyrhiza* removed between 9 and 19% [15]. For *E. crassipes* in the same study, efficiencies of 12.7; 17.7; 19, and 28.2% were reported for fluoride concentrations of 3, 5, 10, and 20 mg L^−1^, respectively, in a 10-day exposure period. Thus, efficiencies reached herein when *E. crassipes* plants were exposed to 15 mg L^−1^ were greater than aforementioned ones (49.4–61.8%, Table 6). Among 10 plant species tested, fluoride removals varied from 51 to 98% when the starting concentration was 5 mg L^−1^ [16]. *Nerium oleander* performed the best and went through further evaluation, presenting remarkable removal rates of 92, 81, 71, 60, and 51% when starting concentrations were 10, 20, 30, 40, and 50 mg L^−1^, respectively.

Overall, *E. crassipes* may be considered for fluoride removal from water due to the accumulation potential and to a prominent growth rate [36]. It is important to underline though that accumulation rates did not represent the whole defluoridation process, as fluoride escaping from solution potentially occurred through other pathways as well (e.g., precipitation, evaporation, and adsorption) [37,54].

## 5. Conclusions

The higher the fluoride initial concentration, the greater the mass of fluoride absorbed by *Eichhornia crassipes*, though in relative terms such removals remained around 60%. These removals mostly occurred on the first 6 days of exposure. Fluoride initial concentration presented the greatest effect on response variables and showed potential for even higher fluoride removals for values above the ones tested in this research. So, a topic for future evaluations is to work this variable individually, expanding the interval of concentrations to find a point at which increments on fluoride absorption by *E. crassipes* can no longer be seen due to phytotoxic effects of the excess of this contaminant.

Auxin amendments via solution in concentrations varying from 1.4 to 3 µM provided greater fluoride removals per dry mass of plants in acid to neutral pH conditions, while the spraying of gibberellin (GA_3_) did not present effects. Phosphorus contributed to higher defluoridation rates, since this element is important to plant nutrition and development and it plays a role regarding the precipitation of fluoride ions as well.

Fluoride contents in *E. crassipes* tissues were higher than other reports, indicating that adding exogenous auxin potentially contributed to greater fluoride removals. Accumulation occurred mostly in aerial parts of the plants. Finally, the giant duckweed *E. crassipes* may be considered as a great candidate for the phytoremediation of fluoride in water, and auxin adding can be a strategy to improve plant tolerance and enhance fluoride removals. The costs of taking such measures, however, are yet to be explored.

## Figures and Tables

**Table 1:** Values of auxin (IAA), gibberellin (GA_3_), pH, phosphorus, and fluoride initial concentration for the definitive screening design experiment.

		Coded Levels	
Variable	−1	0	+1
IAA (µM)	0	5	10
GA_3_ (µM)	0	25	50
pH	5	7	9
P (mg L^−1^)	2.14	4.28	6.42
F (mg L^−1^)	5	10	15

**Table 2:** Treatments generated for the definitive screening design experiment.

	Coded Values					Decoded Values *				
Treatment	IAA	GA_3_	pH	P	F	IAA	GA_3_	pH	P	F
1	0	1	1	1	1	5	50	9	6.42	15
2	0	−1	−1	−1	−1	5	0	5	2.14	5
3	1	0	1	−1	−1	10	25	9	2.14	5
4	−1	0	−1	1	1	0	25	5	6.42	15
5	1	1	0	1	−1	10	50	7	6.42	5
6	−1	−1	0	−1	1	0	0	7	2.14	15
7	1	−1	1	0	1	10	0	9	4.28	15
8	−1	1	−1	0	−1	0	50	5	4.28	5
9	1	−1	−1	1	0	10	0	5	6.42	10
10	−1	1	1	−1	0	0	50	9	2.14	10
11	1	1	−1	−1	1	10	50	5	2.14	15
12	−1	−1	1	1	−1	0	0	9	6.42	5
13	0	0	0	0	0	5	25	7	4.28	10

* Units for each variable can be checked at Table 1.

**Table 3:** Values of auxin (IAA), pH and phosphorus concentration for the central composite design (α = 1.682).

	Coded Levels				
Variable	−α	−1	0	1	α
IAA (µM)	0	0.51	1.25	1.99	2.50
pH	5	5.8	7	8.2	9
P (mg L^−1^)	1.00	2.82	5.50	8.18	10.00

**Table 4:** Treatments generated for the central composite design.

	Coded Values			Decoded Values *		
Treatment	IAA	P	pH	IAA	P	pH
1	−1	−1	−1	0.51	2.82	5.81
2	1	−1	−1	1.99	2.82	5.81
3	−1	1	−1	0.51	8.18	5.81
4	1	1	−1	1.99	8.18	5.81
5	−1	−1	1	0.51	2.82	8.19
6	1	−1	1	1.99	2.82	8.19
7	−1	1	1	0.51	8.18	8.19
8	1	1	1	1.99	8.18	8.19
9	−α	0	0	0.00	5.50	7.00
10	α	0	0	2.50	5.50	7.00
11	0	−α	0	1.25	1.00	7.00
12	0	α	0	1.25	10.00	7.00
13	0	0	−α	1.25	5.50	5.00
14	0	0	α	1.25	5.50	9.00
15	0	0	0	1.25	5.50	7.00
16	0	0	0	1.25	5.50	7.00
17	0	0	0	1.25	5.50	7.00
18	0	0	0	1.25	5.50	7.00

* Units for each variable can be checked at Table 3.

**Table 5:** Removal of fluoride in mg ($F_{rem}$), percentage ($F_{rem}(\%)$), and per mass of plant ($F_{rem/plant}$, mg g^−1^) for the definitive screening design experiment.

Treat.	IAA	GA_3_	pH	P	F	$F_{rem}$ (mg)	$F_{rem}$ (%)	$F_{rem/plant}$ (mg g^−1^)
1	5	50	9	6.42	15	16.47 (±0.96) *	54.9	12.45 (±0.59) *
2	5	0	5	2.14	5	5.73 (±0.24)	57.3	1.96 (±0.07)
3	10	25	9	2.14	5	5.73 (±0.63)	57.2	0.98 (±0.05)
4	0	25	5	6.42	15	16.49 (±1.40)	54.9	13.86 (±0.96)
5	10	50	7	6.42	5	5.88 (±0.40)	58.8	1.20 (±0.06)
6	0	0	7	2.14	15	18.90 (±1.20)	63.0	6.72 (±0.35)
7	10	0	9	4.28	15	18.44 (±0.85)	61.5	5.54 (±0.21)
8	0	50	5	4.28	5	6.17 (±0.48)	61.7	1.74 (±0.11)
9	10	0	5	6.42	10	12.37 (±0.31)	61.8	5.79 (±0.15)
10	0	50	9	2.14	10	12.51 (±0.83)	62.5	9.27 (±0.50)
11	10	50	5	2.14	15	18.40 (±0.88)	61.3	5.22 (±0.29)
12	0	0	9	6.42	5	5.95 (±0.80)	59.5	8.88 (±0.39)
13	5	25	7	4.28	10	11.89 (±0.29)	59.4	2.73 (±0.08)

* Values in parenthesis represent standard deviation from three replicates.

**Table 6:** Removal of fluoride in mg ($F_{rem}$), percentage ($F_{rem}(\%)$) and per mass of plant ($F_{rem/plant}$, mg g^−1^) for the central composite design experiment.

Treat.	IAA	pH	P	$F_{rem}$ (mg)	$F_{rem}$ (%)	$F_{rem/plant}$ (mg g^−1^)
1	0.51	5.8	2.82	17.24 (±1.22) *	57.5	4.03 (±0.28) *
2	1.99	5.8	2.82	15.27 (±0.31)	50.9	6.42 (±0.10)
3	0.51	5.8	8.18	16.24 (±0.38)	54.1	4.46 (±0.09)
4	1.99	5.8	8.18	16.07 (±0.67)	53.6	5.12 (±0.41)
5	0.51	8.2	2.82	15.62 (±0.33)	52.1	5.14 (±0.12)
6	1.99	8.2	2.82	16.51 (±0.81)	55.0	4.86 (±0.24)
7	0.51	8.2	8.18	16.35 (±0.35)	54.5	4.91 (±0.10)
8	1.99	8.2	8.18	15.68 (±0.69)	52.3	4.26 (±0.29)
9	0.00	7.0	5.50	16.17 (±0.71)	53.9	4.89 (±0.21)
10	2.50	7.0	5.50	15.91 (±0.51)	53.0	6.17 (±0.20)
11	1.25	7.0	1.00	16.21 (±0.53)	54.0	4.60 (±0.32)
12	1.25	7.0	10.00	14.82 (±0.48)	49.4	4.99 (±0.26)
13	1.25	5.0	5.50	15.65 (±0.50)	52.2	6.58 (±0.21)
14	1.25	9.0	5.50	15.37 (±0.79)	51.2	4.82 (±0.25)
15	1.25	7.0	5.50	16.70 (±1.07)	55.7	5.49 (±0.55)
16	1.25	7.0	5.50	15.78 (±0.59)	52.6	5.89 (±0.22)
17	1.25	7.0	5.50	15.79 (±0.21)	52.6	4.67 (±0.12)
18	1.25	7.0	5.50	18.54 (±0.38)	61.8	5.47 (±0.30)

* Values in parentheses represent standard deviation from two replicates.

**Table 7:** Fluoride contents in plant tissues ${\left[F^{-}\right]}_{aerial}$ and ${\left[F^{-}\right]}_{root}$, in mg g^−1^, dry weight), bioaccumulation factor (BF) (Equation (4)), and translocation factor (TF) (Equation (5)) of *E. crassipes* exposed to 15 mg L^−1^ of fluoride for 10 days.

Treatment	Aux	pH	P	${\left[F^{-}\right]}_{aerial}$	${\left[F^{-}\right]}_{root}$	BF	TF
1	0.51	5.8	2.82	0.624 (±0.06) *	0.498 (±0.02) *	0.201	1.252
2	1.99	5.8	2.82	0.235 (±0.02)	0.359 (±0.02)	0.042	0.655
3	0.51	5.8	8.18	0.421 (±0.05)	0.374 (±0.01)	0.109	1.125
4	1.99	5.8	8.18	0.523 (±0.02)	0.327 (±0.01)	0.113	1.599
5	0.51	8.2	2.82	0.490 (±0.02)	0.347 (±0.02)	0.099	1.411
6	1.99	8.2	2.82	0.796 (±0.04)	0.423 (±0.03)	0.187	1.882
7	0.51	8.2	8.18	0.538 (±0.04)	0.306 (±(0.01)	0.120	1.756
8	1.99	8.2	8.18	0.326 (±0.03)	0.344 (±0.02)	0.085	0.946
9	0.00	7.0	5.50	0.513 (±0.04)	0.415 (±0.02)	0.119	1.237
10	2.50	7.0	5.50	0.529 (±0.08)	0.374 (±0.01)	0.091	1.413
11	1.25	7.0	1.00	0.518 (±0.06)	0.479 (±0.01)	0.130	1.080
12	1.25	7.0	10.00	0.377 (±0.05)	0.396 (±0.02)	0.074	0.952
13	1.25	5.0	5.50	0.775 (±0.04)	0.326 (±0.02)	0.116	2.374
14	1.25	9.0	5.50	0.521 (0.02)	0.353 (±0.01)	0.109	1.473
15	1.25	7.0	5.50	0.635 (±0.05)	0.375 (±0.02)	0.138	1.696
16	1.25	7.0	5.50	0.379 (±0.04)	0.413 (±0.04)	0.072	0.917
17	1.25	7.0	5.50	0.613 (±0.02)	0.321 (±0.01)	0.136	1.911
18	1.25	7.0	5.50	0.386 (±0.05)	0.451 (±0.05)	0.118	0.857

* Values in parentheses represent standard deviation from two replicates.

## Data Availability

The data that support the findings of this study are available from the corresponding author upon reasonable request.
